# Unilateral Optic Neuritis Post-COVID-19 Infection: A Case Report

**DOI:** 10.7759/cureus.101003

**Published:** 2026-01-07

**Authors:** Aaron D'Amore, Lachlan Driver, Imikomobong Ibia, Paul Chen

**Affiliations:** 1 Emergency Medicine, Mass General Brigham, Harvard Medical School, Boston, USA; 2 Emergency Medicine, Brown University Health, Providence, USA; 3 Emergency Medicine, Children's National Hospital, Washington, DC, USA; 4 Emergency Medicine, Brigham and Women’s Hospital, Harvard Medical School, Boston, USA

**Keywords:** autoimmune, covid-19, neuro-ophthalmology, optic neuritis, sars-cov-2, steroids, vision loss

## Abstract

COVID-19 has affected millions of individuals worldwide, yet the neuro-ophthalmic consequences among survivors remain incompletely characterized. In this case report, we describe a case of unilateral optic neuritis identified in the emergency department using ultrasound in a previously healthy young woman shortly after confirmed SARS-CoV-2 infection. This case highlights the importance of recognizing neuro-ophthalmic sequelae of COVID-19, outlines the diagnostic evaluation, and demonstrates clinical improvement with corticosteroid therapy.

## Introduction

The coronavirus disease 2019 (COVID-19) pandemic, caused by severe acute respiratory syndrome coronavirus 2 (SARS-CoV-2), has been associated with a broad range of systemic complications, including neurologic and ocular manifestations. While pulmonary and cardiovascular sequelae are well described, neuro-ophthalmic complications remain less well understood [1].

Optic neuritis is a condition that causes acute inflammatory demyelination of the optic nerve. The condition typically presents with subacute vision loss, dyschromatopsia, pain with ocular mobility, and a relative afferent pupillary defect. The condition is classically linked to multiple sclerosis (MS) and neuromyelitis optica spectrum disorder (NMOSD), though viral infections are also commonly recognized triggers [2].

MS, the most common disabling neurologic disorder in young adults, frequently produces asymptomatic lesions in non-eloquent brain regions such as the frontal or temporal lobes. However, involvement of eloquent structures such as the optic nerve or brainstem can result in characteristic syndromes, including optic neuritis and internuclear ophthalmoplegia [3]. Histopathology of optic neuritis closely resembles that of acute MS plaques, with perivascular inflammatory cuffing, edema within myelinated nerve sheaths, and extensive myelin breakdown that exceeds axonal loss. In some cases, retinal vascular endothelial inflammation manifests as retinal vein sheathing [4].

The disease is believed to be immune-mediated, though the precise mechanism and antigenic targets remain uncertain. Systemic T-cell activation is typically evident at symptom onset and precedes changes in the cerebrospinal fluid (CSF). Peripheral activation normalizes within weeks, while local inflammation persists, driven by T-cell-derived cytokines and other mediators. B-cell responses against myelin proteins are often detectable in the CSF, but not in peripheral blood [5,6].

Twenty percent of patients have been noted to take three months or more to recover from the effects of the COVID-19 infection [7]. We present a case of unilateral optic neuritis occurring in the post-COVID recovery period in a previously healthy young woman, highlighting diagnostic challenges and management considerations.

## Case presentation

A 31-year-old woman with no significant past medical history presented with 10 days of progressive left-sided sphenoid sinus pressure, headache, and blurry vision. She had a confirmed COVID-19 infection by PCR one month prior to her ED presentation.

**Figure 1:**
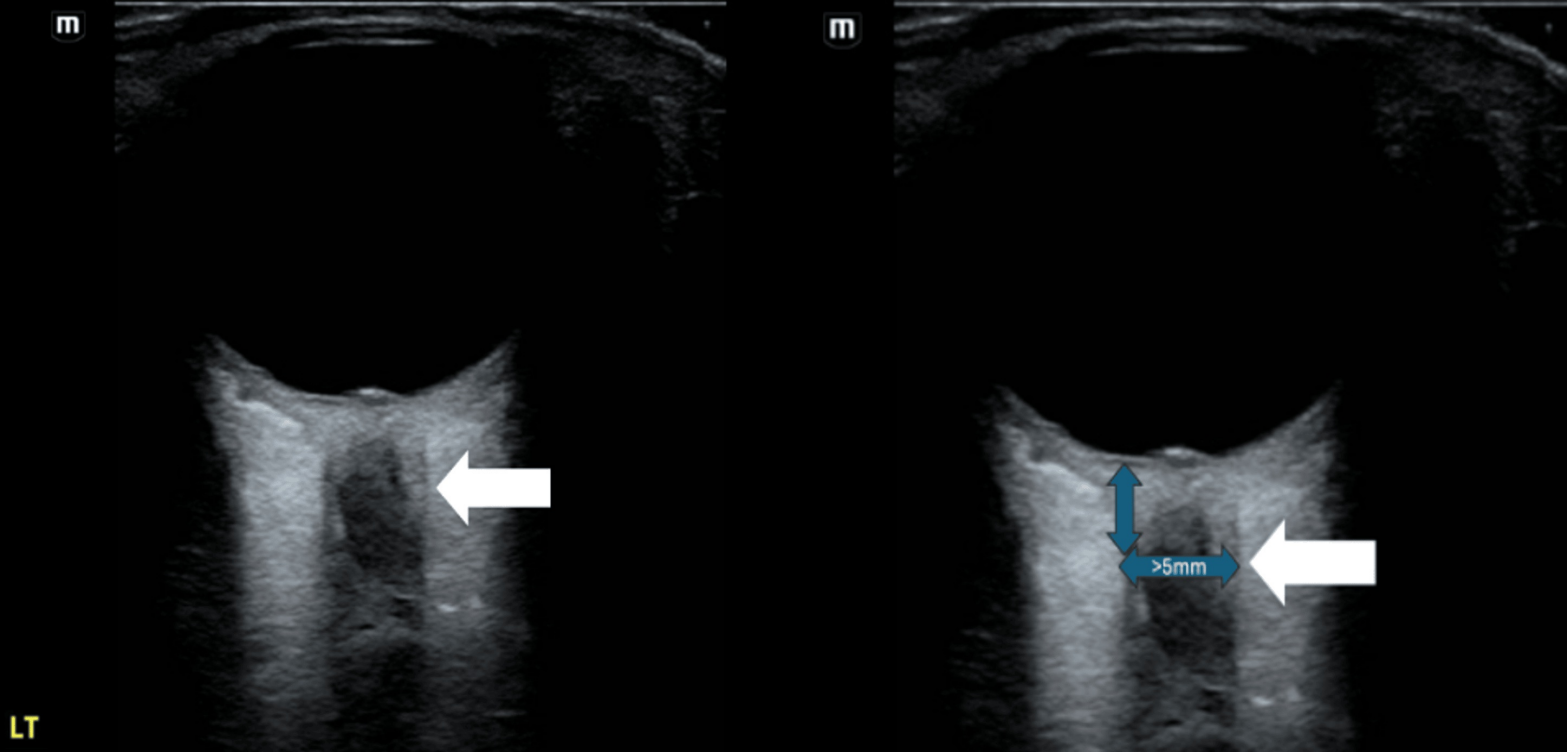
Bedside ocular ultrasound of the left eye demonstrating an abnormal optic nerve consistent with optic neuritis (white arrow).

**Figure 2:**
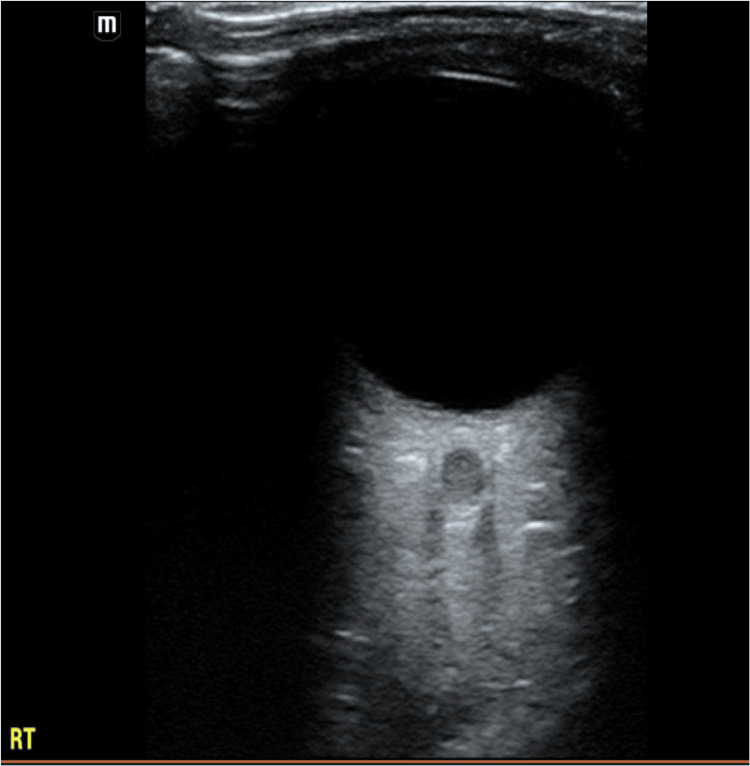
Bedside ocular ultrasound of the right eye showing a normal optic nerve for comparison.

On arrival, she was afebrile and hemodynamically stable. Visual acuity was 20/50 in the right eye and 20/160 in the left eye. Bedside ocular ultrasound was performed using a high-frequency linear transducer with the patient supine and eyes closed. A generous amount of gel was applied over the eyelid. The optic nerve sheath diameter (ONSD) was measured 3 mm posterior to the globe in the transverse and sagittal planes. Reference value <5.0 mm is considered normal in adults [8]. In this patient, the left ONSD was enlarged (>5.0 mm) compared with the right, consistent with optic nerve pathology (Figures 1, 2). The left eye demonstrated dyschromatopsia (1/8 Ishihara plates correct), a brisk relative afferent pupillary defect, and moderate discomfort with extraocular movements. Fundus examination revealed Frisen grade 2 disc edema with mild hyperemia and no hemorrhages. Visual field testing showed partial inferior temporal and nasal defects.

Laboratory studies were obtained as outlined in Table 1 and revealed normal inflammatory markers without evidence of systemic infection. Autoimmune testing revealed a positive ANA; all other serologies were negative.

**Table 1:** Lab studies obtained for the patient compared to typical reference values. ESR: erythrocyte sedimentation rate; CRP: C-reactive protein; WBC: white blood cell count; Hct: hematocrit; Plts: platelets; ANA: antinuclear antibody; IFA: indirect immunofluorescence assay; ANCA: antineutrophil cytoplasmic antibodies; MOG IgG: myelin oligodendrocyte glycoprotein immunoglobulin G; CBA: cell-based assay; NMO (AQP4) IgG: neuromyelitis optica aquaporin-4 immunoglobulin G; ACE: angiotensin-converting enzyme; ELISA: enzyme-linked immunosorbent assay; AI: antibody index; IgG/IgM: immunoglobulin G and immunoglobulin M.

Test	Patient's Value	Typical Reference Range
ESR (mm/hr)	4	0–20
CRP (mg/dL)	0.4	<1.0
WBC (×10³/µL)	10.3	4.0–11.0
Hct (%)	32.3	36–44
Plts (×10³/µL)	148	150–400
ANA (IFA)	Positive (≥1:40)	<1:40
ANCA (U/mL)	Negative (<20)	<20
MOG IgG (CBA)	Negative (<1:20)	<1:20
NMO (AQP4) IgG (AI)	Negative (<1.5)	<1.5
ACE level (U/L)	Normal (<40)	9–67
Lyme ELISA (AI)	Negative (<0.9)	<0.9
Syphilis IgG/IgM antibody (AI)	Negative (<0.9)	<0.9

Magnetic resonance imaging (MRI) of the brain and orbits revealed unilateral left optic neuritis without evidence of other demyelinating lesions.

The patient was treated with intravenous methylprednisolone 1 g daily for three days and admitted to the inpatient neurology floor for monitoring. At her follow-up appointment, visual acuity in her left eye had improved to 20/15, and the relative afferent pupillary defect had resolved. Dyschromatopsia persisted but was mild. Visual field testing was full in the right eye and revealed only a small cecocentral scotoma in the left eye. Optical coherence tomography demonstrated resolution of disc edema with interval development of mild optic atrophy and partial macular ganglion cell layer thinning.

Overall, the findings suggested post-infectious optic neuritis related to SARS-CoV-2 infection. The optic nerve enhancement measured 2.6 cm which is longer than what is typically seen with MS-associated optic neuritis [3]. Optic nerve enhancement of this length is more characteristic of anti-myelin oligodendrocyte glycoprotein antibody-associated disease (MOGAD) or NMSOD, though antibody testing for anti-MOG and anti-NMO was negative [9]. The patient was scheduled for repeat MRI brain and orbits at six months to assess the future risk of MS. While six-month follow-up MRI was never obtained, the patient did represent to the ED approximately four years later with blurry vision in the right eye. Repeat MRI was completed at that visit, which identified right-sided optic neuritis and resolved left-sided optic neuritis with mild left-sided optic nerve atrophy. It also identified few small, chronic appearing, and nonspecific white matter foci, not typical for demyelinating disease. 

## Discussion

COVID-19 has been documented in the literature to be associated with systemic neurologic signs in as many as 21% and cranial nerve impairment in about 0.4% of infected individuals [1]. Several case reports have suggested a temporal relationship between SARS-CoV-2 infection and optic neuritis. Patients presented with symptoms including subacute vision loss, dyschromatopsia, pain with eye movement, and a relative afferent pupillary defect [3,10-13]. In our case, the temporal proximity of infection to symptom onset, lack of prior neurologic disease, and negative serologic workup suggest a post-infectious immune-mediated mechanism. 

Neuro-ophthalmic complications of COVID-19 described in the literature include optic neuritis, ischemic optic neuropathy, cranial nerve palsies, and uveitis [3,10-13]. This is thought to occur as a result of an immunologic response to the peripheral nerve antigens, resulting in demyelination and nerve injury, though the exact mechanisms remain under investigation [1]. Proposed mechanisms include direct viral invasion of neural tissue, systemic T-cell activation with cytokine release, and vascular injury related to endothelial dysfunction or hypercoagulability [14,15]. In our case, the positive ANA raised the possibility of unmasking an autoimmune predisposition, though the clinical course favored a post-viral etiology. Our case is especially unique, given that the patient presented several years later with right-sided blurry vision and was found to have optic neuritis in the right eye as well as non-specific white matter lesions not typical for demyelinating disease.

High-dose corticosteroids remain standard treatment for optic neuritis, hastening visual recovery though not altering long-term outcomes [16]. Our patient’s rapid improvement mirrors findings in prior reports of post-COVID optic neuritis managed with steroids. The long segment of optic nerve enhancement seen on MRI is more typical of NMOSD or MOGAD, but negative antibody testing and a favorable prognosis further support a post-infectious pathogenesis [9].

This case contributes to the growing body of literature describing immune-mediated optic neuritis after SARS-CoV-2 infection and underscores the need for ongoing surveillance of neuro-ophthalmic complications in post-COVID patients.

Teaching points

Clinicians should maintain a high index of suspicion for optic neuritis in patients presenting with acute visual changes after documented COVID-19 infection. Clinical features of this include subacute vision loss, dyschromatopsia, pain with eye movement, and a relative afferent pupillary defect. It is outlined in our case how the use of point-of-care ocular ultrasound (POCUS) can rapidly detect optic nerve abnormalities at the bedside and help differentiate optic neuritis from other causes of vision loss. When using POCUS for this, it is important to understand that ONSD should be measured 3 mm posterior to the globe on POCUS. Values >5 mm in adults are considered abnormal and may support the diagnosis of optic neuritis or elevated intracranial pressure [8]. MRI of the brain and orbits, along with antibody testing for MOG and NMO, is essential to differentiate infectious, autoimmune, and demyelinating etiologies. The gold standard therapy of optic neuritis is high-dose corticosteroids. These medications have been proven to accelerate the recovery of vision but do not change long-term prognosis.

## Conclusions

This case adds to the growing evidence that SARS-CoV-2 infection may precipitate immune-mediated neuro-ophthalmic complications, including optic neuritis. The patient’s clinical course, negative infectious and autoimmune evaluation, and rapid response to corticosteroids support a post-infectious inflammatory mechanism rather than a primary demyelinating disorder such as MS, NMOSD, or MOGAD. Although isolated case reports such as this one cannot establish causality, the temporal association between COVID-19 infection and symptom onset, combined with increasingly recognized reports of similar presentations, underscores the need for clinicians to maintain a high index of suspicion for optic neuritis when evaluating patients with visual complaints in the post-COVID-19 infection period.

As the long-term effects of SARS-CoV-2 continue to unfold, heightened awareness of neuro-ophthalmic sequelae is essential. Bedside ocular ultrasound, when used in conjunction with MRI and comprehensive serologic testing, may serve as a valuable adjunct in early diagnosis, particularly in emergency care settings. Prompt initiation of high-dose corticosteroids remains the cornerstone of treatment and can significantly improve short-term visual outcomes, as demonstrated in this case. Further research is needed to clarify the underlying immunopathogenesis linking SARS-CoV-2 infection to optic nerve inflammation, to determine whether certain individuals harbor latent autoimmune susceptibility unmasked by viral infection, and to characterize the long-term risk of future demyelinating disease. Ongoing surveillance and reporting of post-COVID-19 neuro-ophthalmic presentations will be critical to defining the true spectrum of disease. Ultimately, this case highlights the complex interplay between infection and autoimmunity and reinforces the importance of recognizing and treating optic neuritis promptly to preserve visual function in patients recovering from COVID-19.

## References

[1] Leung EH, Fan J, Flynn HW Jr, Albini TA (2022). Ocular and systemic complications of COVID-19: impact on patients and healthcare. Clin Ophthalmol.

[2] Golnik KC (2002). Infectious optic neuropathy. Semin Ophthalmol.

[3] Frohman EM, Frohman TC, Zee DS, McColl R, Galetta S (2005). The neuro-ophthalmology of multiple sclerosis. Lancet Neurol.

[4] Lightman S, McDonald WI, Bird AC, Francis DA, Hoskins A, Batchelor JR, Halliday AM (1987). Retinal venous sheathing in optic neuritis. Its significance for the pathogenesis of multiple sclerosis. Brain.

[5] Roed H, Frederiksen J, Langkilde A, Sørensen TL, Lauritzen M, Sellebjerg F (2005). Systemic T-cell activation in acute clinically isolated optic neuritis. J Neuroimmunol.

[6] Söderström M, Link H, Xu Z, Fredriksson S (1993). Optic neuritis and multiple sclerosis: anti-MBP and anti-MBP peptide antibody-secreting cells are accumulated in CSF. Neurology.

[7] Oelsner EC, Sun Y, Balte PP (2024). Epidemiologic features of recovery from SARS-CoV-2 infection. JAMA Netw Open.

[8] Ballantyne SA, O’Neill G, Hamilton R, Hollman AS (2002). Observer variation in the sonographic measurement of optic nerve sheath diameter in normal adults. Eur J Ultrasound.

[9] Ciotti JR, Eby NS, Brier MR, Wu GF, Chahin S, Cross AH, Naismith RT (2022). Central vein sign and other radiographic features distinguishing myelin oligodendrocyte glycoprotein antibody disease from multiple sclerosis and aquaporin-4 antibody-positive neuromyelitis optica. Mult Scler.

[10] Gluckstein JA, Chwalisz BK, Gilbert AL, Bouffard MA (2023). SARS-CoV-2 parainfectious optic neuropathy: 3 case reports and a review of the literature. J Neuroophthalmol.

[11] Azab MA, Hasaneen SF, Hanifa H, Azzam AY (2021). Optic neuritis post-COVID-19 infection. A case report with meta-analysis. Interdiscip Neurosurg.

[12] Jossy A, Jacob N, Sarkar S, Gokhale T, Kaliaperumal S, Deb AK (2022). COVID-19-associated optic neuritis - a case series and review of literature. Indian J Ophthalmol.

[13] Zhang J, Joiner D, Zhang C (2023). Hyperacute optic neuritis in a patient with COVID-19 infection and vaccination: a case report. BMC Ophthalmol.

[14] Golabchi K, Rezaee A, Aghadoost D, Hashemipour M (2021). Anterior ischemic optic neuropathy as a rare manifestation of COVID-19: a case report. Future Virol.

[15] Alomari SO, Abou-Mrad Z, Bydon A (2020). COVID-19 and the central nervous system. Clin Neurol Neurosurg.

[16] Beck RW, Cleary PA, Anderson MM Jr (1992). A randomized, controlled trial of corticosteroids in the treatment of acute optic neuritis. The Optic Neuritis Study Group. N Engl J Med.

